# Elevated TAF12 Expression Predicts Poor Prognosis in Glioma Patients: Evidence from Bioinformatic and Immunohistochemical Analyses

**DOI:** 10.3390/biom12121847

**Published:** 2022-12-10

**Authors:** Xiaodi Guo, Jiamin Chen, Aizhong Fang, Qiang Ji, Feng Chen, Xingang Zhou, Xinyi Li, Wenbin Li

**Affiliations:** 1 Department of Neuro-Oncology, Neurosurgery Center, Beijing Tiantan Hospital, Capital Medical University, Beijing 100070, China; 2 Department of Oncology, Beijing Ditan Hospital, Capital Medical University, Beijing 100015, China; 3 Department of Pathology, Beijing Ditan Hospital, Capital Medical University, Beijing 100015, China; 4 Department of Nursing School, University of South Florida, Tampa, FL 33620, USA

**Keywords:** TAF12, glioma, bioinformatics, prognosis, immune

## Abstract

TATA box-binding protein-associated factor 12 (TAF12) has been identified as an oncogene in choroid plexus carcinoma, but its role in glioma is poorly understood because of a lack of previous studies. This study investigated the relationship of TAF12 expression with the clinicopathologic features of glioma cases, as well as its prognostic value and biological function, using large-scale databases and clinical samples. TAF12 mRNA expression and clinicopathologic characteristics of glioma cases were assessed in three public databases, and bioinformatics analyses were conducted to explore the prognostic value and biological functions of TAF12 in glioma. High TAF12 expression was commonly associated with reduced survival time and poor clinical indexes, including higher World Health Organization grade, wild-type isocitrate dehydrogenase 1 expression, and 1p19q non-codeletion status (*p* < 0.0001). Multivariate Cox regression analysis showed that high TAF12 expression was an independent poor prognostic factor for glioma patients (hazard ratio = 1.41, 95% confidence interval, 1.18–1.68, *p* < 0.001). Functional enrichment analysis revealed involvement of TAF12 in immune and inflammatory responses in glioma. Also, expression of several immune checkpoint molecules was significantly higher in samples with high TAF12 expression. TAF12 is a potential independent prognostic factor for glioma, and these findings provide a foundation for further investigation of the potential role of TAF12 in immunotherapy.

## 1. Introduction

Gliomas are the most common and lethal intrinsic malignant tumors in the central nervous system (CNS) and account for 25.1% of all primary brain and other CNS tumors in the United States [1]. Despite advancements in neurosurgery and radiotherapy for glioma treatment, the median observed survival time for glioma, especially high-grade glioma patients, remains short due to the aggressiveness of tumors, drug resistance, and recurrence [2]. In recent years, wild-type isocitrate dehydrogenase 1 (IDH1) mutation, 1p/19q codeletion, and O6-methylguanine-DNA methyltransferase (MGMT) promoter methylation have been widely applied for molecular classification of glioma, but these markers are of limited value for predicting prognosis and response to therapy. Therefore, a better understanding of the molecular mechanisms underlying tumorigenesis in glioma cases is urgently needed to support the development of new strategies for disease monitoring, treatment, and prognosis evaluation.

The general transcription factor IID (TFIID), which is a mega-Dalton sized complex containing TATA box-binding protein (TBP) and many conserved TBP-associated factors (TAFs), recognizes and binds promoter DNA to engage RNA polymerase II and several relevant factors for transcription [3,4]. Although the mechanism by which TFIID recognizes the promoter remains incompletely understood, previous research has raised the possibility that TBP might be key in this process. TAFs, as the TBP-associated factor subunits, are thought to play several important roles in transcription, such as recognizing downstream promotor elements, acting as coactivators, and interacting with nucleosomes [3]. Recently, TBP-associated factor 12 (TAF12) has been shown to be important for the RAS-induced transformation properties of human colon cells [5] and identified as an oncogene in choroid plexus carcinoma [6].To date, studies on the role of TAF12 in glioma have been very limited. Ren et al. using gene over-expression and gene-chip techniques, found mutated IDH1 down regulates TAF12 expression in U87 cell lines [7]. Wijethilake et al. reported the high expression of TAF12 with six other genes is associated with poor survival of glioma patients, based on novel probabilistic programming [8]. However, they did not show the expression pattern and potential functions of TAF12 in glioma.

To further investigate the potential role of TAF12 in glioma in the present study, we first examined the transcriptional level of TAF12 and its relationship with the clinicopathological characteristics of glioma cases using data from multiple public databases and our clinical specimens. We then performed bioinformatics analyses to explore the biological function and prognostic value of TAF12 in glioma.

## 2. Materials and Methods

### 2.1. Dataset Selection

We first investigated the association between TAF12 gene expression and glioma clinical characteristics using data from three large public databases. The TAF12 mRNA expression level and relevant clinical data in glioma cases were obtained from The Cancer Genome Atlas (TCGA) dataset as a discovery set and Chinese Glioma Genome Atlas (CGGA) dataset [9] and Gene Expression Omnibus GSE16011 database [10] as validation sets. Additionally, we performed immunohistochemistry (IHC) on glioma samples from 28 glioma patients treated in the Department of Neurosurgery, Ditan Hospital (Beijing, China) to confirm the results of bioinformatics analysis.

### 2.2. Data Downloading and Preprocessing/Data Acquisition

The mRNAseq data for 698 gliomas, ranging from grade II to grade IV according to 2016 World Health Organization (WHO) classification of CNS tumors, and 5 normal brain tissues were downloaded from TCGA databases (https://portal.gdc.cancer.gov/ (assessed on 10 August 2020)). Gene RNA sequencing and corresponding clinical data were downloaded for 693 glioma samples from the mRNAseq_693 dataset and 325 glioma samples from the mRNAseq_325 dataset (http://www.cgga.org.cn (assessed on 10 August 2020)). The gene expression data from the two datasets were corrected in batches and integrated by loading into the “sva [11]” and “limma [12]” packages in R software v3.6.3. The GSE16011 dataset contained 276 glioma samples and 8 normal control samples (http://www.ncbi.nlm.nih.gov/geo/query/acc.cgi?acc=GSE16011 (assessed on 21 May 2022)).

### 2.3. Immunohistochemistry

After surgical excision, tumor tissues were placed immediately in 10% formalin for fixation, followed by dehydration, paraffin embedding, and sectioning. Slides were then stained with TAF12 rabbit polyclonal antibody (1:1000, Proteintech, IL, USA ) overnight at 4 °C and then incubated in secondary antibody solution for 1 h at room temperature. The results were confirmed by two experienced neuropathologists independently. TAF12 protein expression (semi-quantitative scoring) was equal to expression intensity multiplied by expression area. Expression intensity was scored using a 4-point scale from 0 to 3, and expression area was scored using a 5-point scale from 0 to 4. The results for TAF12 expression were then categorized based on score ranges of 0–6, 7–9 and 10–12, which were represented by +, ++ and +++, respectively [13].

### 2.4. Bioinformatics Analysis

The differential expression of TAF12 in tumor versus normal tissues was analyzed using data from TCGA and GSE16011, and Gene Expression Profiling Interactive Analysis (GEPIA) was performed using TCGA and GTEx data [14]. In three public datasets, correlations between TAF12 expression and various clinicopathologic characteristics were analyzed and printed using the “ggpubr” and “beeswarm” R packages. Kaplan–Meier survival analysis, univariate and multivariate Cox analyses, and receiver operating characteristic (ROC) curve analysis were performed using the “survival”, “survminer”, and “survivalROC” R packages. Six was used as the cut-off value of Kaplan Meier survival analysis on the IHC samples based on semi-quantitative scoring of TAF12 protein expression.

Differentially expressed genes (DEGs) were identified by applying the Wilcoxon test in comparisons between high- and low-expression groups with a false discovery rate (FDR) < 0.05 and |logFC| > 1. DEGs were explored using the “clusterProfiler [15]” package in R for gene ontology (GO) and Kyoto Encyclopedia of Genes and Genomes (KEGG) analyses. Gene set enrichment analysis (GSEA) 4.1.0 [16] was conducted to confirm the differential enrichment of various biological processes and signaling pathways between the high- and low-expression groups. The annotated gene sets from c2.cp.kegg.v7.4.symbols.gmt were adopted in the molecular signatures database (MSigDB) [17]. A normal *p* < 0.05 and FDR *q* < 0.25 were used as thresholds.

### 2.5. Analysis of Immune Response

The single-sample GSEA (ssGSEA) was used to analyze the RNA-sequencing (RNA-seq) data of 29 important immune gene sets [18] from each glioma sample in the form of ssGSEA scores using the R packages “GSVA [19]”, “limma”, and “GSEABase”. According to the estimation of the numbers of stromal and immune cells in malignant tumor tissues using the expression data (ESTIMATE) [20] algorithm, we calculated the tumor purity, ESTIMATE score, immune score, and stromal score for each glioma sample using the “estimate” R package. Then, the “pheatmap” R package was used to display the clustering heatmap of immune-related gene scores between the two TAF12 expression groups. In addition, the relationships between 22 types of tumor-infiltrating immune cells (TIICs) in the glioma microenvironment and the TAF12 expression level were assessed using the CIBESORT (cell-type identification by estimating relative subsets of RNA transcripts) deconvolution algorithm [21].

### 2.6. Meta-Analysis

Meta-analysis was performed to evaluate the overall prognostic value of TAF12 expression in glioma patients among three datasets. The hazard ratio (HR) and 95% confidence interval (CI) were the principal measures. Data analysis was performed using the “meta” R package. Heterogeneity among the datasets was quantified using a Q test and *I*^2^ statistics. If the *I*^2^ value was <50%, a fixed-effects model was used; otherwise, a random-effects model was used [22].

### 2.7. Statistical Analysis

Patients with missing information were excluded from the corresponding analysis. The correlations between mRNA expression and clinicopathologic characteristics were analyzed using the Mann–Whitney U test for two groups or the Kruskal–Wallis test for more than two groups. Semi-quantitative scores from IHC analysis were analyzed using the Mann–Whitney U test. Correlations were evaluated using Spearman correlation analysis. Kaplan–Meier survival analysis was used to evaluate the overall survival of glioma patients with high and low TAF12 expression. The differences between groups were assessed by log-rank test. These statistical analyses were performed utilizing R software v.3.6.3 and GraphPad Prism 9. The R packages used were downloaded from CRAN and Bioconductor for data processing data and figure preparation. All statistical tests were two-sided, and *p* < 0.05 was used as the cutoff for statistical significance.

## 3. Results

### 3.1. Characteristics of Patients

A total of 2033 samples were included in this study from three public datasets and Ditan dataset. The characteristics of these cases is shown in Table 1.

### 3.2. Transcriptional Levels of TAF12 Were Upregulated in Glioma

**Figure 1:**
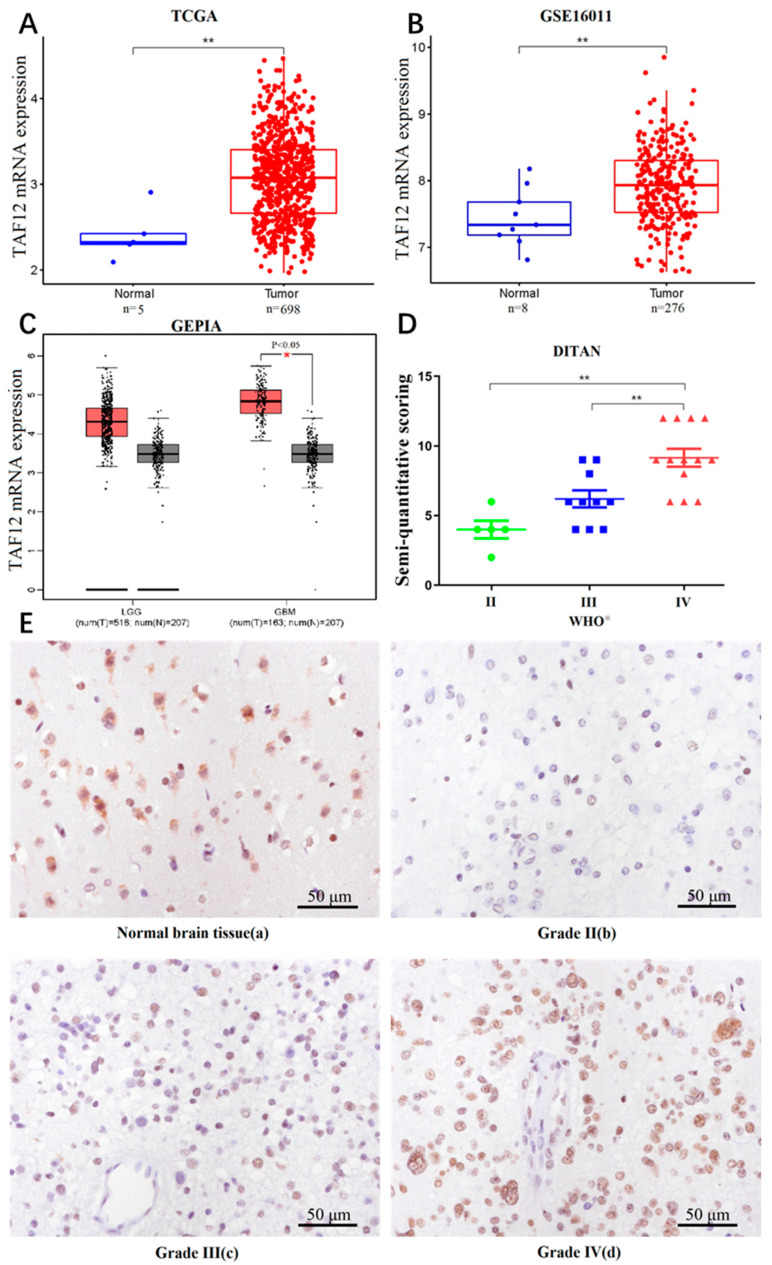
mRNA and protein expression levels of TAF12 in glioma versus normal samples. (**A**,**B**) TAF12 mRNA expression was upregulated in glioma compared with normal brain tissues (** *p* ≤ 0.01). (**C**) Based on GEPIA (match TCGA normal and GTEx data), TAF12 was also upregulated in GBM (* *p* ≤ 0.05). (**D**) TAF12 protein expression in glioma samples from Beijing Ditan Hospital by IHC (** *p* ≤ 0.01). (**E**) IHC staining showing TAF12 protein expression in glioma samples of different WHO grades. (**a**) Positive control from cerebral cortex of tumor-adjacent tissues, showing strong TAF12 expression in the cytoplasm and nuclei of neurons. (**b**) Weak positive expression of TAF12 in WHO Ⅱ glioma sample. (**c**) Moderate positive expression of TAF12 in WHO Ⅲ glioma sample. (**d**) Strong positive expression of TAF12 in WHO Ⅳ glioma sample (EnVision; original magnification, ×400). ※ According to 4th WHO classification (WHO 2016 classification).

We compared the transcriptional levels of TAF12 in glioma samples with those in normal samples from TCGA and GSE16011. The results from the two datasets demonstrated that TAF12 was significantly upregulated in glioma samples compared with normal samples (Figure 1A,B). Furthermore, the mRNA expression level of TAF12 was also apparently higher in glioblastoma multiforme (GBM) and relatively increased in low-grade glioma (LGG) compared with normal samples from TCGA and GTEx datasets based on GEPIA analysis (Figure 1C).

To verify these results, we randomly selected 28 patients for IHC analysis to detect the correlation between tumor grade and TAF12 protein expression. Semi-quantitative scoring of IHC staining showed that TAF12 was enriched in high-grade glioma (HGG), and TAF12 protein expression was positively associated with WHO grade (Figure 1D,E).

### 3.3. TAF12 Expression Is Associated with Glioma Grade, Subtype and Molecular Features

**Figure 2:**
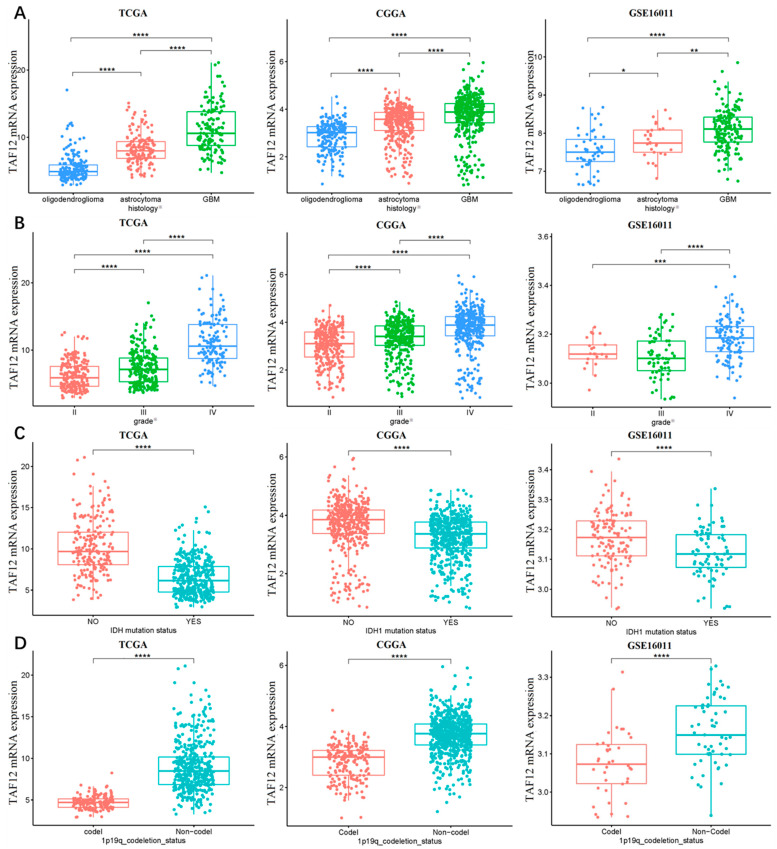
Associations between TAF12 mRNA expression and molecular characteristics of glioma among cases in three different datasets. Associations between TAF12 mRNA expression and: (**A**) histological type; (**B**) WHO grade; (**C**) IDH1 mutation status; and (**D**) 1p19q codeletion status. * *p* ≤ 0.05, ** *p* ≤ 0.01, *** *p* ≤ 0.001 and **** *p* ≤ 0.0001. ※ According to 4th WHO classification (WHO 2016 classification).

Due to the histopathological heterogeneity of glioma, the TAF12 mRNA expression data were analyzed according to WHO grade, histology, IDH1 mutation, and other features. In the three included datasets, TAF12 expression was the highest in the GBM compared with other lower histopathologic malignancies (Figure 2A). Additionally, we found that TAF12 expression was positively correlated with tumor grade (Figure 2B). In the 2016 CNS WHO criteria, the IDH1 genotypes and status of 1p and 19q codeletion were absorbed into the classification of gliomas, and therefore, we investigated the distribution of TAF12 expression in the above molecular subclasses of gliomas. TAF12 was highly expressed in cases with wild-type IDH1 and 1p19q non-codeletion. Additionally, although higher TAF12 expression was observed in patients older than 40 years in the CGGA and TCGA, this difference was not confirmed in the GEO datasets (Supplementary Figure S1A). Moreover, although previous studies reported that MGMT methylation was associated with a better prognosis and better response to chemotherapy in GBM patients [23], lower TAF12 expression was observed in samples with MGMT methylation in the CGGA (Supplementary Figure S1B). Furthermore, because of an update to the classification system, we also evaluated the expression level of TAF12 according to the grading of adult diffuse gliomas in the 2021 5th edition of the WHO classification of tumors of the CNS. The results showed that TAF12 expression was also different among three groups (Supplementary Figure S1C). Together these findings suggest that the high TAF12 expression can predict a high level of malignancy in glioma.

### 3.4. High TAF12 Expression Predicts Poor Prognosis in Glioma Patients

**Figure 3:**
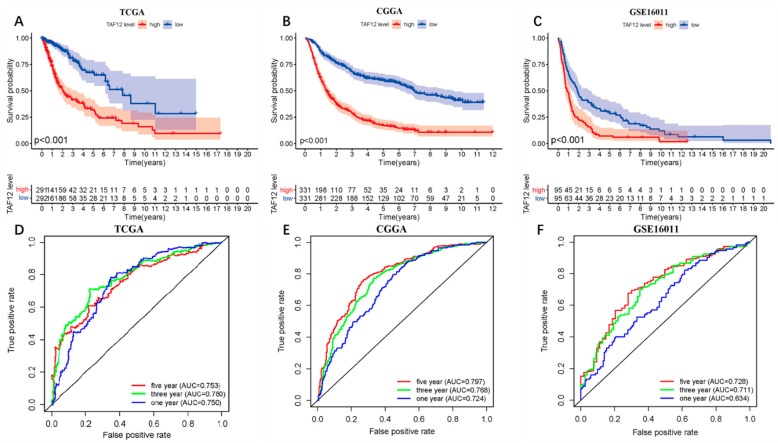
Association of TAF12 expression with the prognosis of glioma patients in different datasets and treated at Beijing Ditan Hospital. (**A**–**C**) Glioma patients with higher expression of TAF12 had a shorter overall survival time. (**D**–**F**) Time-dependent ROC curves for overall survival (OS) at 1, 3, and 5 years.

To evaluate the prognostic value of TAF12 expression, samples were divided into low- or high-expression groups based on the median value. Kaplan–Meier survival analysis demonstrated that higher TAF12 expression was correlated with shorter overall survival of glioma patients in both the discovery and validation sets (Figure 3A–C). ROC curve analysis of samples from the TCGA showed that TAF12 expression could predict 1-, 3-, and 5-year survival (AUC = 0.750, 0.780, and 0.753, respectively; Figure 3D). The prognostic value of TAF12 expression for gliomas was further confirmed in the two validation sets (Figure 3E,F). Additionally, the quantitative IHC results for TAF12 protein expression in our patient samples provided similar results, with patients in the group with higher TAF12 protein expression having a significantly worse prognosis (Supplementary Figure S2). Together, these findings demonstrated that TAF12 expression was a negative prognostic factor in glioma patients.

### 3.5. TAF12 Is an Independent Prognostic Factor for Glioma Patients

**Figure 4:**
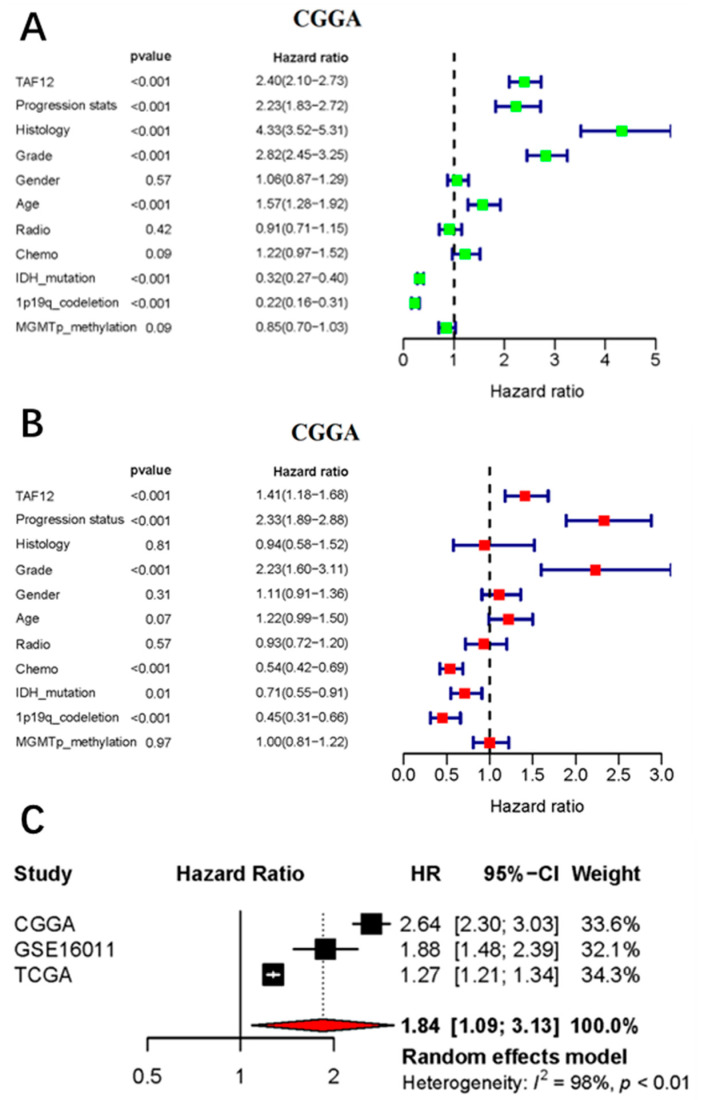
Cox regression analysis and meta-analysis of factors with prognostic significance in glioma. (**A**,**B**) Univariate and multivariate Cox analyses of factors affecting the overall survival of glioma patients in the CGGA database. (**C**) Forrest plot from meta-analysis of the association of high TAF12 expression with worse overall survival in glioma patients from three databases.

To further examine the prognostic value of TAF12 expression, we performed univariate Cox regression analysis using data from the CGGA database first. The results showed that patients with high TAF12 expression had worse overall survival (HR = 2.40, 95%CI 2.10–2.73, *p* < 0.001), and many other clinicopathologic parameters (primary-recurrent-secondary (PRS) type, histology, grade, age, IDH1 status, and 1p19q codeletion status) also showed significant correlations with overall survival (Figure 4A). Next, multivariate Cox regression analysis was performed and identified high TAF12 expression (HR = 1.41, 95%CI 1.18–1.68, *p* < 0.001), recurrence (HR = 2.33, 95%CI 1.89–2.88, *p* < 0.001). and higher WHO grade (HR = 2.23, 95%CI 1.60–3.11, *p* < 0.001) as independent factors associated with a poor prognosis. Conversely, receiving chemotherapy, having the IDH1 mutation, and having 1p/19q codeletion independently showed correlation with improved survival (HR = 0.54, 95%CI 0.42–0.69, *p* < 0.001; HR = 0.71, 95%CI 0.55–0.91, *p* = 0.01; and HR = 0.45, 95%CI 0.31–0.66, *p* < 0.001, respectively; Figure 4B). Finally, meta-analysis of the three datasets confirmed the prognostic role of TAF12 expression in glioma (Figure 4C), indicating that TAF12 may be a novel prognostic biomarker in glioma. The pooled HR was calculated using the random-effects model because of heterogeneity (*I*^2^ = 98%, *p* < 0.01), which may have been, in part, due to the use of different sequencing methods among patients in the three datasets.

### 3.6. TAF12 May Be Involved in the Malignant Progression of Glioma

**Figure 5:**
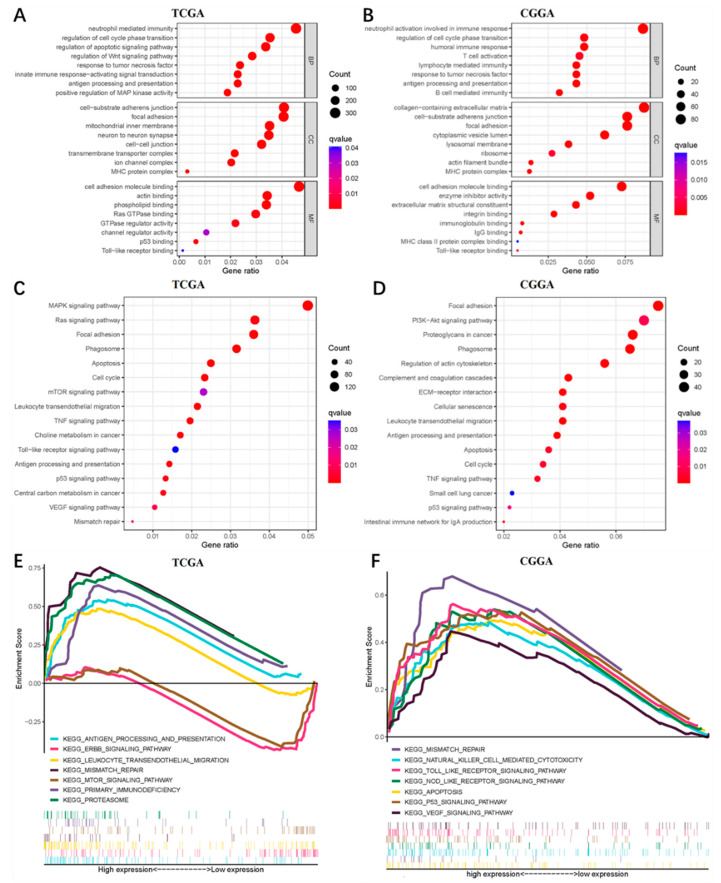
Functional enrichment analysis of TAF12 in glioma. (**A**,**B**) GO analysis of DEGs identified in TCGA and CGGA. (**C**,**D**) KEGG analysis of DEGs identified in TCGA and CGGA. (**E**,**F**) GSEA between low and high TAF12 expression groups in TCGA and CGGA.

To investigate the biological roles of TAF12 in glioma, we first identified DEGs between high and low TAF12 expression groups. From TCGA data we found 7435 DEGs, and from the CGGA data we found 1157 DEGs between these patient groups. We then performed GO and KEGG analyses for these DEGs. GO analysis revealed that DEGs related to TAF12 expression were enriched in the immune and inflammatory responses, cell cycle and cell adhesion (Figure 5A,B). On KEGG pathway analysis, the DEGs related to TAF12 expression were mainly enriched in focal adhesion, leukocyte transendothelial migration, cell cycle, antigen processing and presentation, apoptosis, and some tumor-associated signaling pathways such as the PI3K, Ras, and MAPK pathways (Figure 5C,D). Furthermore, GSEA from the KEGG database showed that high TAF12 expression was also associated with immune-related functions in two databases. Additionally apoptosis, p53 signaling, and vascular endothelial growth factor (VEGF) signaling were found to be enriched in the high TAF12 expression group in the CGGA, and ErbB and mammalian target of rapamycin (mTOR) signaling were enriched in the low TAF12 expression group in TCGA (Figure 5E,F). Together the results of these gene enrichment analyses indicate that TAF12 likely participates in the malignant progression of glioma, particularly in the immune and inflammatory responses in glioma.

### 3.7. TAF12 Is Associated with Immune and Inflammatory Responses in Glioma

**Figure 6:**
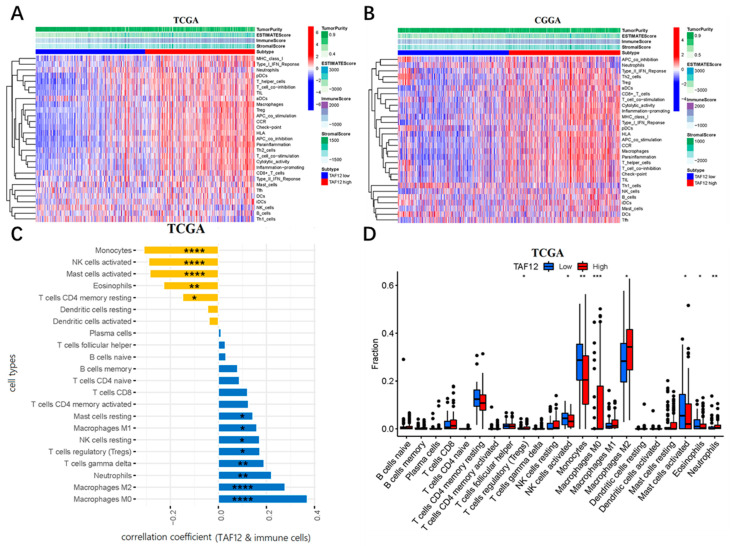
Associations between TAF12 expression and immune cell infiltration in glioma. (**A**,**B**) Heatmaps showing TAF12 expression, tumor immune microenvironment patterns and 29 well-established immune-associated genes based on ssGSEA of TCGA and CGGA data. (**C**) Correlation analysis between TAF12 expression and immune cell infiltration in TCGA samples. Yellow bars indicate a correlation coefficient <0, and blue bars indicate a correlation coefficient >0. (**D**) Comparisons of the abundances of 22 immune cell types in low- and high-TAF12-expression groups from TCGA. * *p* ≤ 0.05, ** *p* ≤ 0.01, *** *p* ≤ 0.001, and **** *p* ≤ 0.0001.

With data from the TCGA and CGGA datasets, ssGSEA was used to explore the relationship between TAF12 expression and immune cell types, as well as immune-related pathways or functions based on the 29 well-established immune-associated gene sets (Supplementary Table S1). Heatmaps showed infiltration of CD8^+^ T cells, macrophages, T helper (Th) cells and tumor-infiltrating lymphocytes (TILs) in glioma samples with high TAF12 expression, whereas fewer Th1_cells showed the opposite trend. Moreover, the immune-related pathways or functions were positively related to TAF12 expression. Marker genes for immune checkpoints, antigen-presenting cell (APC) costimulation, chemokine receptor (CCR) activation, cytolytic activity, pro-inflammatory factors, major histocompatibility complex (MHC) class I molecules, parainflammatory factors, T-cell coinhibition and costimulation, and type I interferon (IFN) response were more highly enriched in samples with high TAF12 expression than those with low TAF12 expression (Figure 6A,B).

As TIICs play a vital pathophysiological role in glioma, we used the CIBERSORT algorithm to analyze the proportions of 22 types of TIICs in TCGA glioma samples. We first assessed the correlation between TAF12 expression and tumor infiltration levels of 22 types of TIICs (Figure 6C). This analysis showed that TAF12 expression was significantly positively correlated with infiltration by M0 and M2 macrophages (R = 0.37 and R = 0.28, respectively, *p* < 0.001), neutrophils (R = 0.22, *p* = 0.002), γδT cells (R = 0.19, *p* = 0.007), regulatory T cells (R = 0.17, *p* = 0.016), resting natural killer (NK) cells (R = 0.17, *p* = 0.016), M1 macrophages (R = 0.16, *p* = 0.024) and resting mast cells (R = 0.14, *p* = 0.042). TAF12 expression also was significantly negatively correlated with infiltration by monocytes (R = −0.31, *p* < 0.001), activated NK cells (R = −0.29, *p* < 0.001), activated mast cells (R = −0.28, *p* < 0.001), eosinophils (R = −0.23, *p* = 0.001) and memory resting CD4 T cells (R = −0.15, *p* = 0.037). Next, we assessed the differences in infiltration by 22 types of immune cells between low and high TAF12 expression subgroups. The high TAF12 expression group showed significantly increased infiltration by M0 macrophages (*p* < 0.001), neutrophils (*p* < 0.01), regulatory T cells (*p* < 0.05) and M2 macrophages (*p* < 0.05), whereas the low TAF12 expression group had relatively enriched infiltration of monocytes (*p* < 0.01), activated NK cells (*p* < 0.05), activated mast cells (*p* < 0.05) and eosinophils (*p* < 0.05).

### 3.8. High TAF12 Expression Is Associated with Greater Sensitivity of Glioma to Immunotherapy

**Figure 7:**
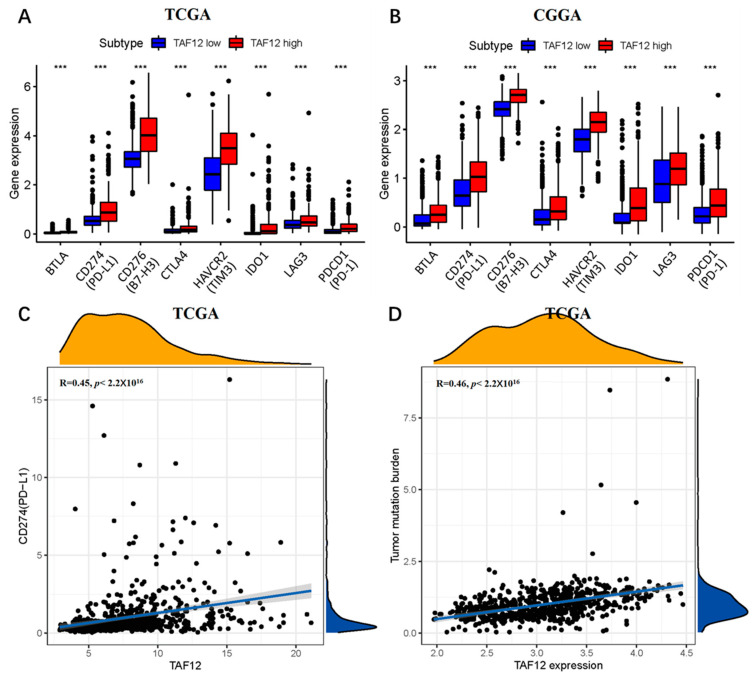
Correlation of TAF12 expression with expression of immune checkpoint molecules and TMB. (**A**,**B**) Expression levels of PD-L1 and other immune checkpoint molecules in high and low TAF12 expression groups in TCGA and CGGA. *** *p* ≤ 0.001. (**C**) Correlation analysis between PD-L1 and TAF12 expression in TCGA. (**D**) Correlation analysis between TMB and TAF12 expression in TCGA.

Immunotherapies such as immune checkpoint inhibitors (ICIs) and chimeric antigen receptor T-cell (CAR-T) therapies have dramatically increased the therapeutic options for multiple cancer types [24]. However, although several trials have explored the usage of immunotherapy against glioma, the results have been unsatisfactory [25], and strategies for the selection of patients most likely to benefit from such therapies are still needed. The intensity of programmed death ligand 1 (PD-L1) expression on tumor cells is one of the most commonly used biomarkers for predicting response to ICIs, and patients with a high TMB are expected to benefit from ICIs. In this study, we assessed the potential of patients with different levels of TAF12 expression to respond to immunotherapies by analyzing the expression levels of several immune checkpoint molecules. Samples with high TAF12 expression from the TCGA and CGGA databases exhibited significantly higher expression levels of PD-L1, B7 homolog 3 (B7-H3), cytotoxic T lymphocyte associated protein 4 (CTLA4), programmed cell death 1 (PD-1), T-cell immunoglobulin and mucin domain 3 (TIM-3), indoleamine-pyrrole 2,3-dioxygenase 1 (IDO1), lymphocyte activation gene 3 (LAG3) and B and T lymphocyte attenuator (BTLA) compared with samples with low TAF12 expression (Figure 7A,B).

Furthermore, we performed a correlation analysis between the expression levels of TAF12 expression and the potential predictors mentioned above. The results showed that TAF12 expression was positively correlated with the expression levels of both PD-L1 and TMB (R = 0.45 and 0.46, respectively, *p* < 0.05; Figure 7C,D). Thus, these immune checkpoint molecules represent possible predictive markers for immunotherapy response in glioma patients.

## 4. Discussion

Gliomas are a group of tumors that usually originate from glial or precursor cells, and GBM is the most malignant type [1]. Unfortunately, advances in conventional cancer therapies and the development of numerous new treatments over the past decades have had very limited effectiveness for improving the prognosis of glioma, especially GBM [2]. Major gaps in our understanding of the molecular mechanisms of glioma pathogenesis and progression have hampered the development of treatment methods. Therefore, there is a critical need to identify biomarkers through microarray technology and bioinformatics analysis that could improve the prognosis of glioma patients.

TAF12 is a component of the TFIID involved in RNA polymerase I and II transcription [26]. Voulgari et al. [5] demonstrated that TAF12 is transcriptionally up-regulated in human colon carcinoma cell lines expressing mutant RAS and actively participates in cellular transformation by influencing E-cadherin expression. Xu et al. [27] showed that TAF12 is a coactivator of MYB that also protects MYB from degradation, and this interaction is mediated by binding between the TAF12/TAF4 histone-fold domain heterodimer and MYB. Their study revealed that loss of TAF12 function could impair MYB activity and cause regression of acute myeloid leukemia (AML) in a mouse model. In addition to its involvement in transcription and role as an oncogene in choroid plexus carcinoma [6], when overexpressed, TAF12 has been reported to cause chromosome instability, which is an inherent enabling characteristic of cancer important for tumor initiation and progression and observed in a majority of tumors [28,29,30].

In the present study, we examined TAF12 gene expression and the clinicopathological characteristics in glioma cases using data from public databases and clinical samples from our institution. We demonstrated that TAF12 was significantly upregulated in glioma samples compared to normal tissues, and high TAF12 expression correlated well with malignant behaviors, such as high WHO grade and IDH wildtype status in glioma. Kaplan–Meier survival analysis showed that high TAF12 expression was associated with shorter overall survival among glioma patients, and multivariate Cox analysis identified TAF12 as an independent poor prognostic factor in patients with glioma. Moreover, ROC curve analysis showed that TAF12 expression could predict 3- and 5-year survival of glioma patients with AUC values >0.7, which supports the use of TAF12 expression as a novel predictor of survival in glioma. Meta-analysis further confirmed the relationship between TAF12 overexpression and worse prognosis in glioma patients. These results are consistent with previous findings [7,8].

Functional enrichment analyses revealed the involvement of TAF12 in a variety of cellular functions and signal regulation pathways related to the malignant progression of glioma, including many immune-related pathways. Previous research has shown that immune cells in the tumor microenvironment, which is a heterogeneous and complex organization composed of tumor, stromal, and endothelial cells, play a critical role in controlling or promoting tumor growth [31]. Here, we found that patients with different levels of TAF12 expression showed significant differences in the proportions of immune-infiltrating cells within glioma samples using ssGSEA and the CIBERSORT algorithm. For example, ssGSEA showed that TAF12 expression was positively correlated with the infiltration of macrophages, and the CIBERSORT algorithm confirmed the proportions of infiltrating M0 and M2 macrophages were higher in the group with high TAF12 expression. Macrophage infiltration is known to be a double-edged sword, as these cells have the potential to both promote and inhibit tumor activity [32]. When tumor-infiltrating macrophages polarize into phenotypically M2 macrophages, they secrete immune-suppressive cytokines and recruit immune-suppressive cells to the tumor microenvironment, which subsequently promote tumorigenesis and metastasis by suppressing immune clearance, promoting tumor cell proliferation and stimulating angiogenesis [33,34]. In contrast, M1 macrophages are known to have anti-tumoral activity and to produce pro-inflammatory cytokines. Our result has also shown that TAF12 expression promotes the infiltration of T regulatory cells, which have key functions in peripheral immune tolerance by helping to balance between disease elimination and the protection of healthy tissues. T regulatory cells [35] modulate the innate and adaptive immune responses by suppressing cytokine production in different subsets of immune cells. NK cells [36] are a specialized immune effector cell type that plays a critical role in immune activation against abnormal cells, but unfortunately, in the present study, the level of activated NK cells in glioma samples was reduced in the high TAF12 expression group. These results strongly indicate that TAF12 play an adverse role in antitumor immunity in glioma.

Immunotherapy, as the fourth pillar of cancer therapy, following surgery, radiotherapy, and chemotherapy, has been extensively investigated in recent years for the treatment of many tumor types, including melanoma and non-small cell lung cancer, and promising results have been reported. Immune checkpoint molecules are expressed on the activated T cell surface and are a type of immunosuppressive molecule that mainly regulate the immune response by T cells to avoid damage to and destruction of normal tissues [37]. Interaction between immune checkpoint molecules and immunosuppressive ligands on tumors leads to immune tolerance during tumor development because anti-tumor T cell activity is suppressed. For this reason, immune checkpoint molecules have become therapeutic targets for immunotherapies. In recent years, several studies in animal models have shown that blockade of immune checkpoint molecules can both enhance the anti-tumor immune response and improve survival [38,39,40]. Unfortunately though, multiple large-scale phase III clinical trials, such as the CHECKMATE 143 [41], 498 [42] and 548 [43] studies, demonstrated no significant survival benefits in patients with both recurrent and naïve GBM without selection. The CNS is well known to be a relatively highly immunosuppressive microenvironment, and thus, gliomas are typically considered “cold tumors”. Additionally, the low TMB of GBM, especially newly diagnosed GBM, is another important reason for its failure to respond to immunotherapy [44]. Bagchi et al. [24] have found that patients with a high TMB may benefit from ICI treatment. Therefore, strategies to identify which patients with a high TMB may benefit from immunotherapy are urgently needed. In the present study, we investigated the correlations between TAF12 expression and several potentially predictive factors for immunotherapy response, including TMB, PD-L1, and classical immune checkpoint molecules. We found that TAF12 expression was positively correlated with both PD-L1 expression and TMB. The expression levels of PD-L1, PD-1, CTLA4, TIM-3, B7-H3, IDO1, LAG3 and BTLA were all significantly increased in tumors with high TAF12 expression. We speculate that TAF12 expression may help to guide personalized patient immunotherapy, even though our current results were obtained merely from public databases, and the precise mechanism of interaction between these factors remains unclear.

Despite the above limitations, this study provides an investigation of the clinicopathological and prognostic significance of TAF12 in glioma. Our findings lay a foundation for further mechanistic studies of TAF12 in glioma. Further in vitro and in vivo experiments examining the molecular functions of TAF12 in glioma will support the development of more accurate prognostic models for patients, which will allow more personalized therapies for gliomas.

## Figures and Tables

**Table 1:** Characteristics of patients in TCGA,CGGA, GSE16011, and Ditan datasets.

Characteristic	TCGA	CGGA	GSE16011	Ditan
Samples	703	1018	284	28
Age (years)				
≤40	236	434	82	8
>40	375	583	194	20
Gender				
Male	357	601	184	15
Female	254	417	92	13
IDH1 status				
mutation	376	531	81	11
wild type	227	435	140	17
1p/19q codeletion status				
codeleted	150	212	45	6
non-codeleted	455	728	73	22
Grade ※				
Ⅱ	214	291	24	5
Ⅲ	237	334	99	10
Ⅳ	160	388	145	13
Histology ※				
non-tumor tissue	5		8	
astrocytoma	167	389	29	9
oligodendroglioma	171	206	52	5
oligoastrocytoma	113	29	28	1
glioblastoma	160	388	159	13
5th WHO classification				
Astrocytoma, IDH-mutant, 1p/19q non-codeleted	219	175	44	5
Oligodendroglioma, IDH-mutant, 1p/19q codeleted	149	121	27	6
Glioblastoma, IDH-wildtype	130	158	78	15

※ According to 4th WHO classification (WHO 2016 classification).

## Data Availability

Publicly available datasets were analyzed in this study.

## Supplementary Materials

The following supporting information can be downloaded at: https://www.mdpi.com/article/10.3390/biom12121847/s1. Supplementary Figure S1: Associations between TAF12 mRNA expression and clinicopathological characteristics of glioma among cases in different datasets. Associations between TAF12 mRNA expression and: (A) age; (B) MGMT methylation status; (C) 5th WHO classification. Supplementary Figure S2: Prognostic significance of TAF12 protein expression as assessed by quantitative IHC analysis.
