# Supplementary figures and images for: Elevated TAF12 Expression Predicts Poor Prognosis in Glioma Patients: Evidence from Bioinformatic and Immunohistochemical Analyses
Source: Biomolecules. 2022 Dec 10;12(12):1847. doi: 10.3390/biom12121847 (PMC9775265; doi:10.3390/biom12121847)

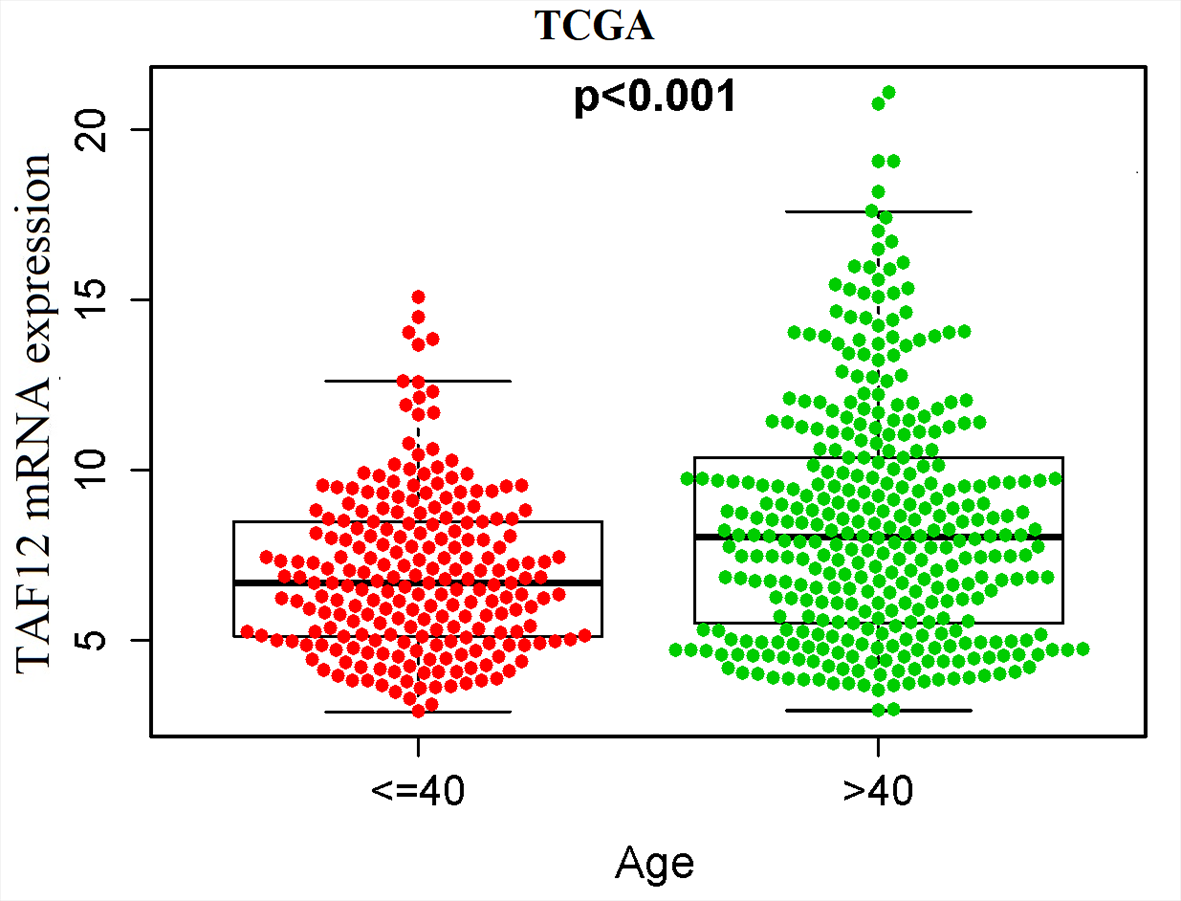

Supplement: Supplementary file 1 [file biomolecules-12-01847-s001.zip › supplementaryfigure S1A1.tif]

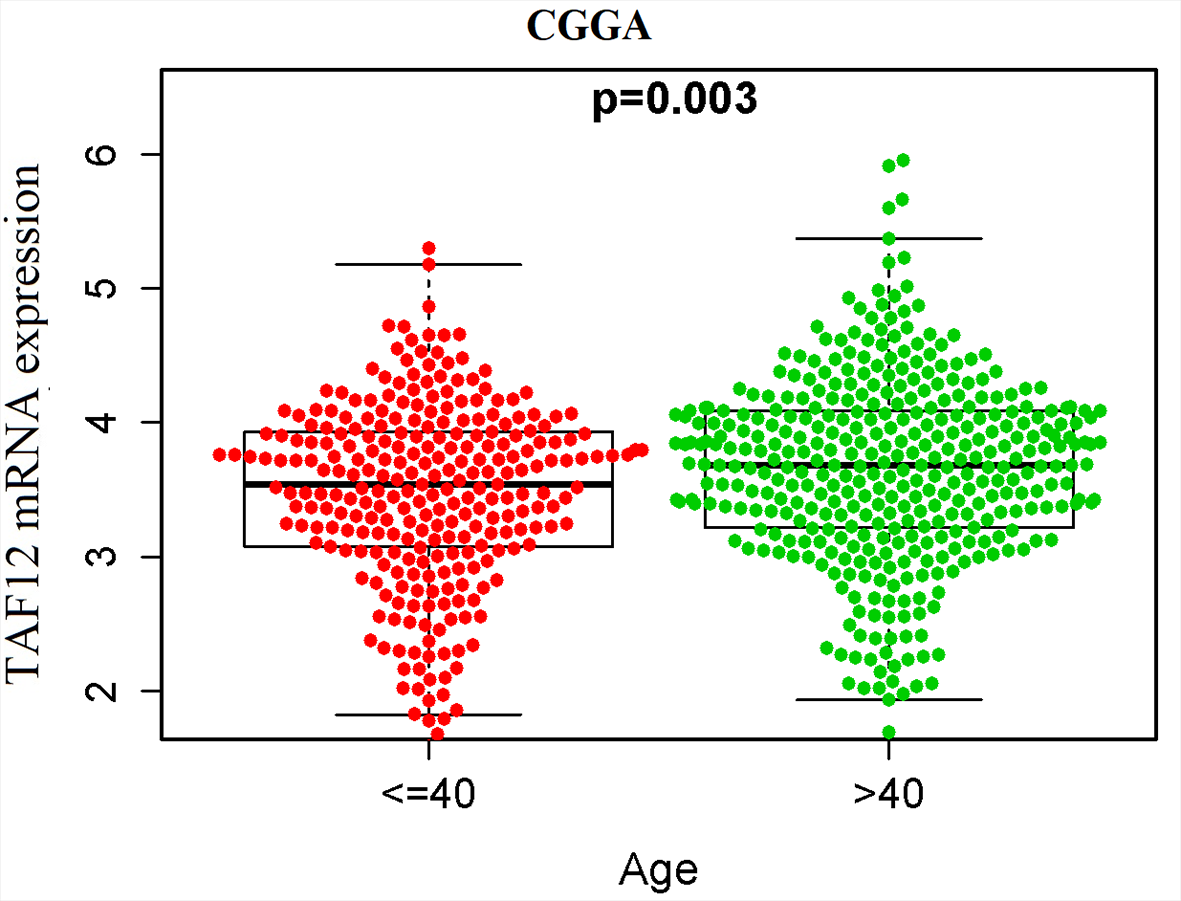

Supplement: Supplementary file 1 [file biomolecules-12-01847-s001.zip › supplementaryfigure S1A2.tif]

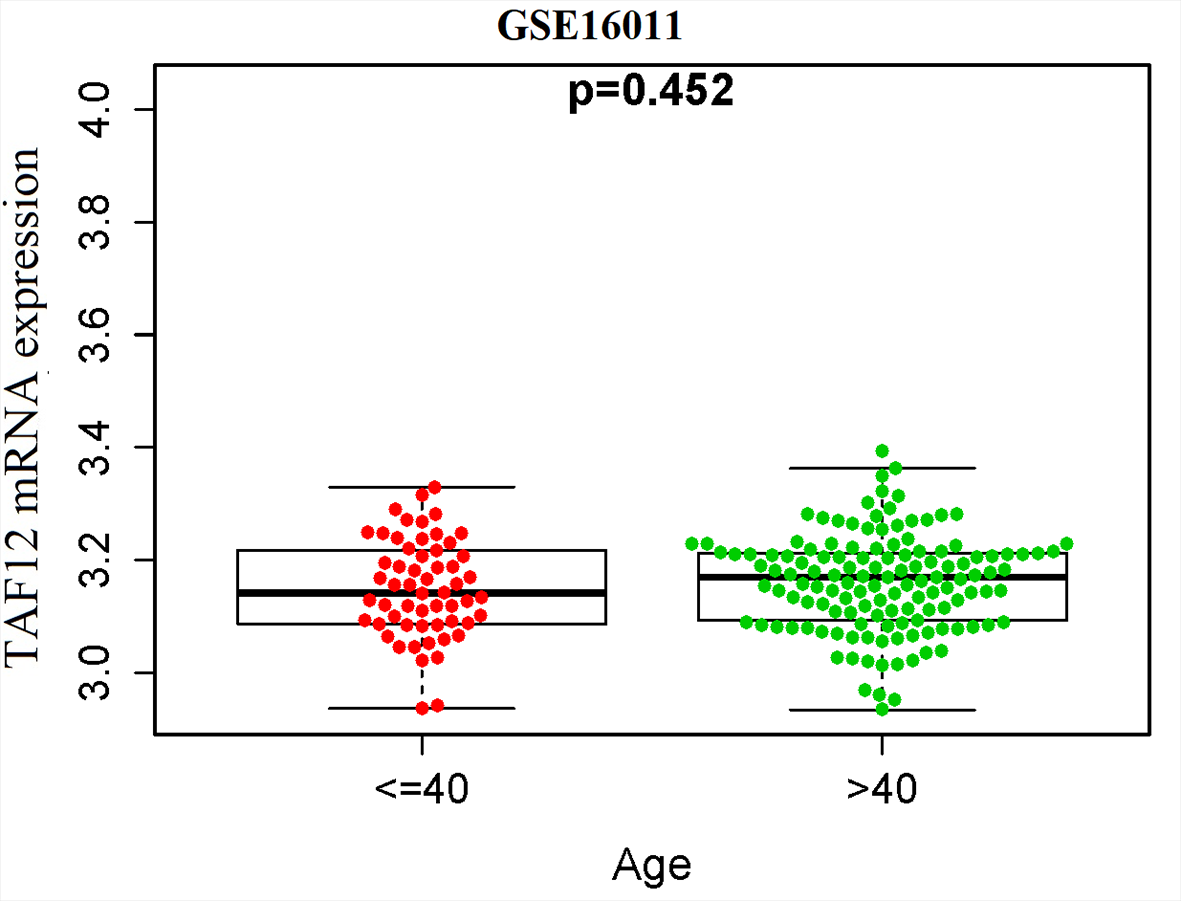

Supplement: Supplementary file 1 [file biomolecules-12-01847-s001.zip › supplementaryfigure S1A3.tif]

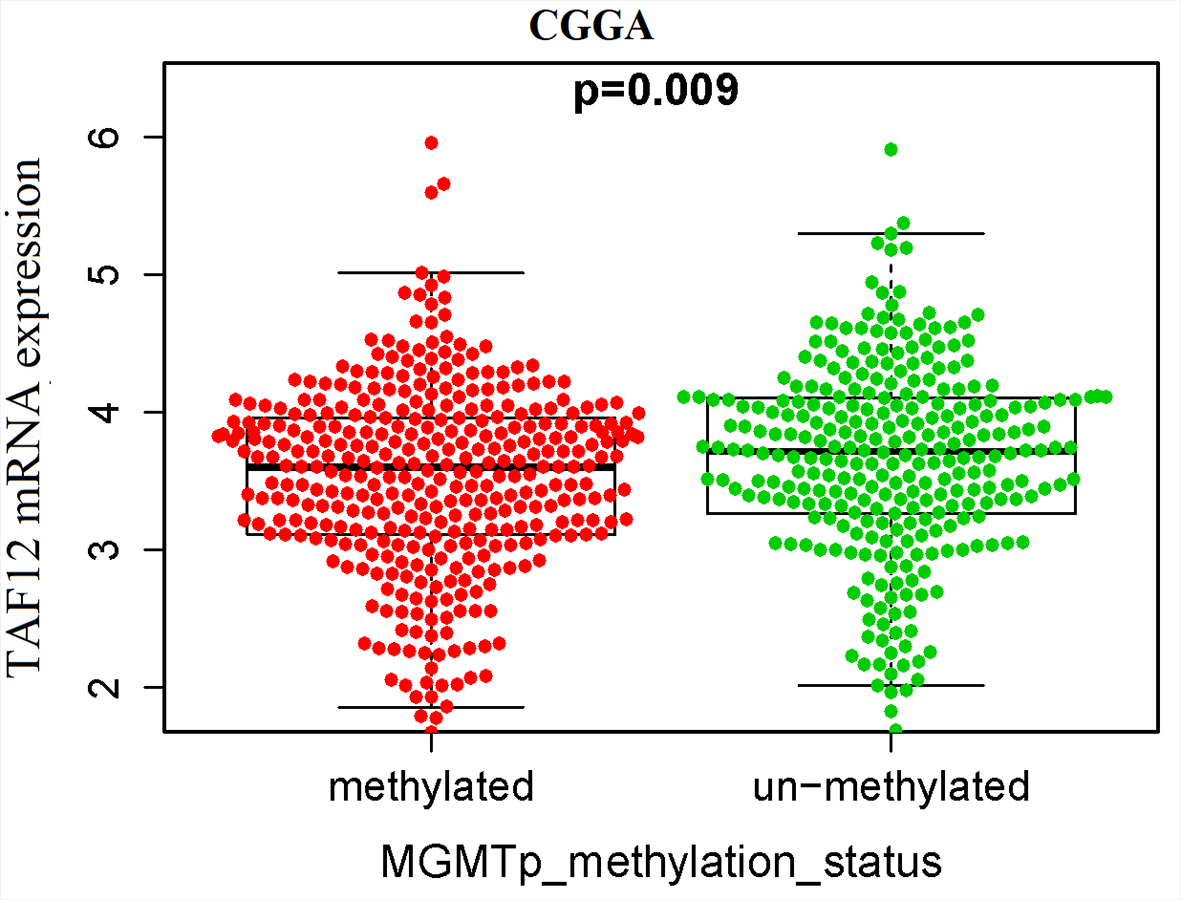

Supplement: Supplementary file 1 [file biomolecules-12-01847-s001.zip › supplementaryfigure S1B.tif]

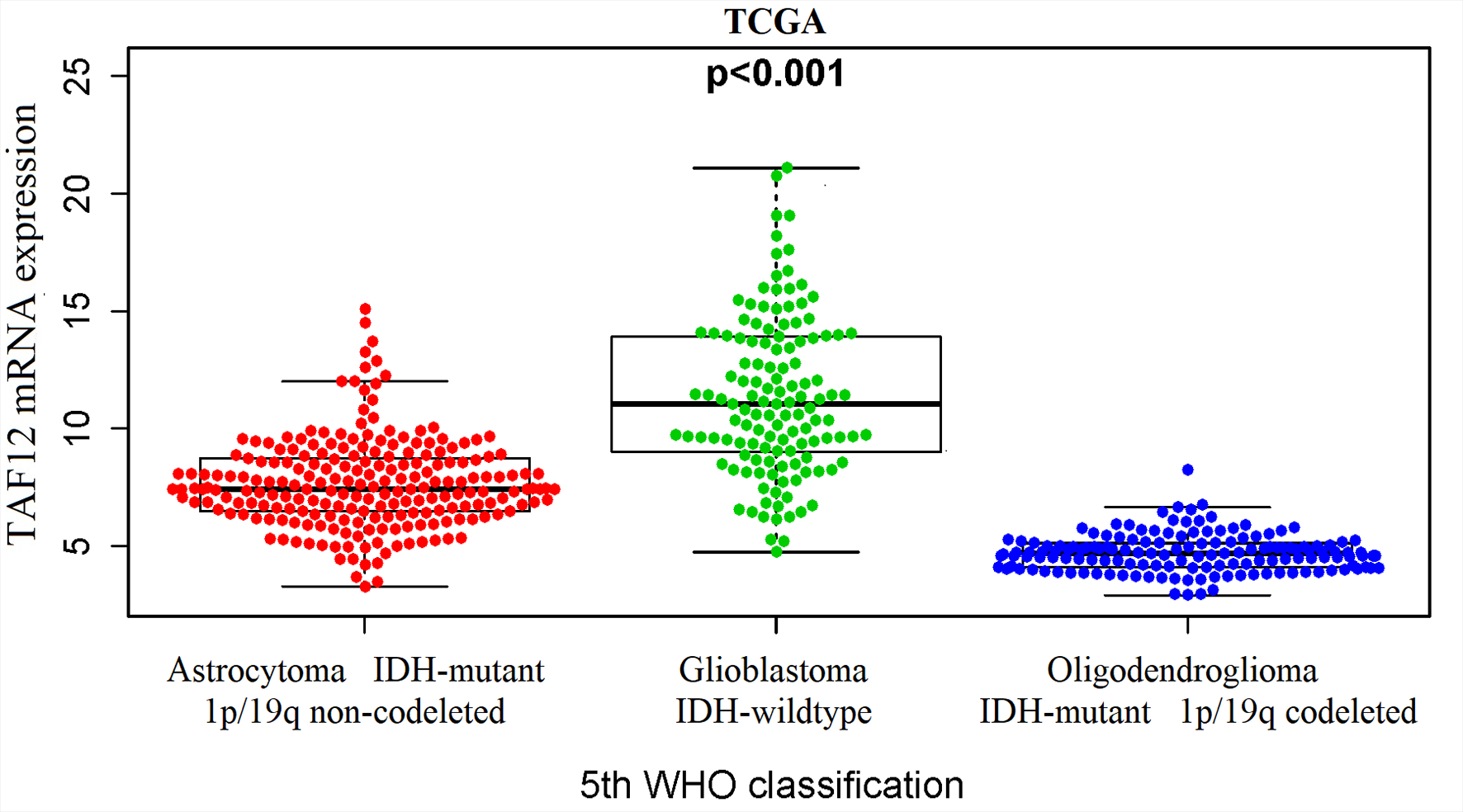

Supplement: Supplementary file 1 [file biomolecules-12-01847-s001.zip › supplementaryfigure S1C1.tif]

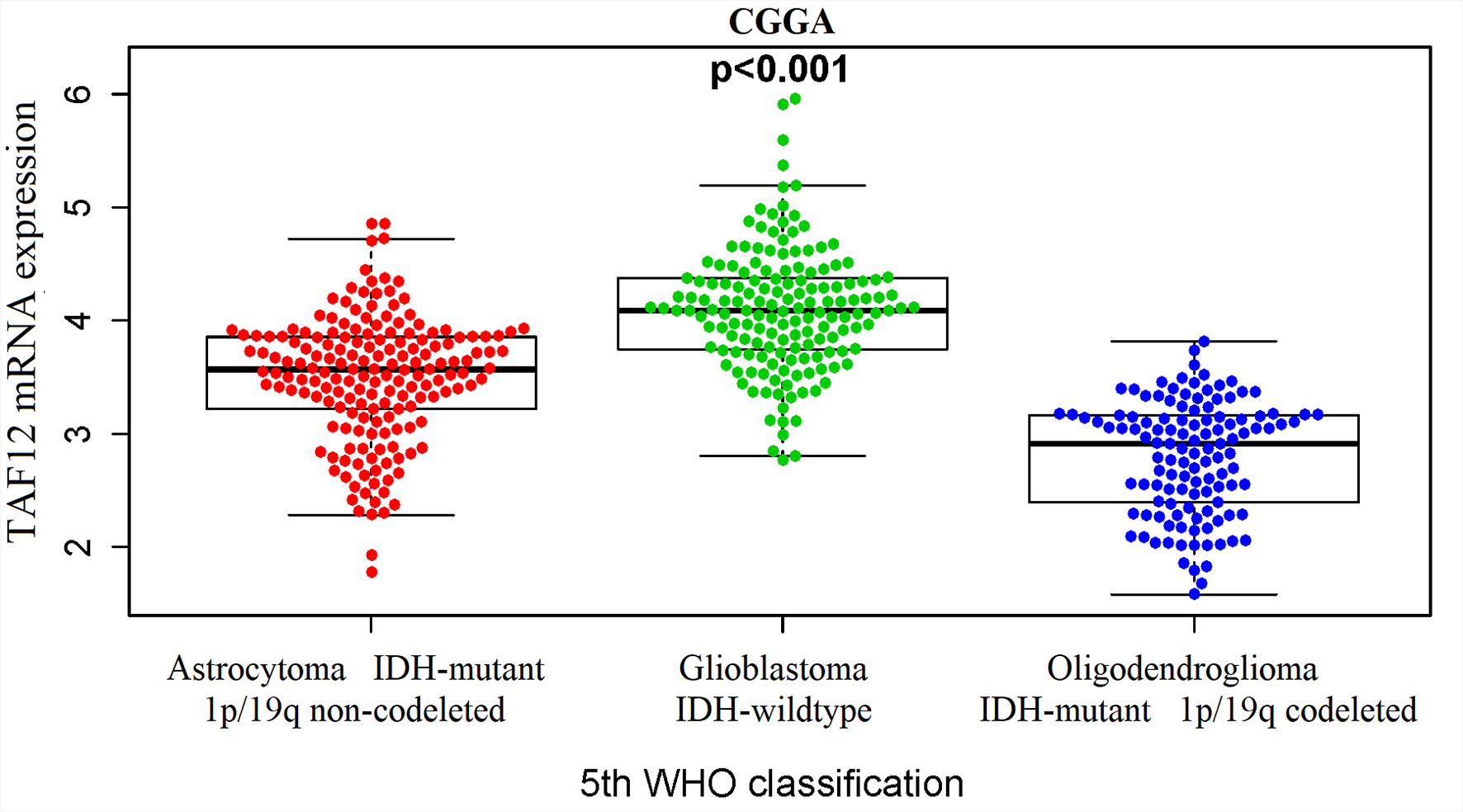

Supplement: Supplementary file 1 [file biomolecules-12-01847-s001.zip › supplementaryfigure S1C2.tif]

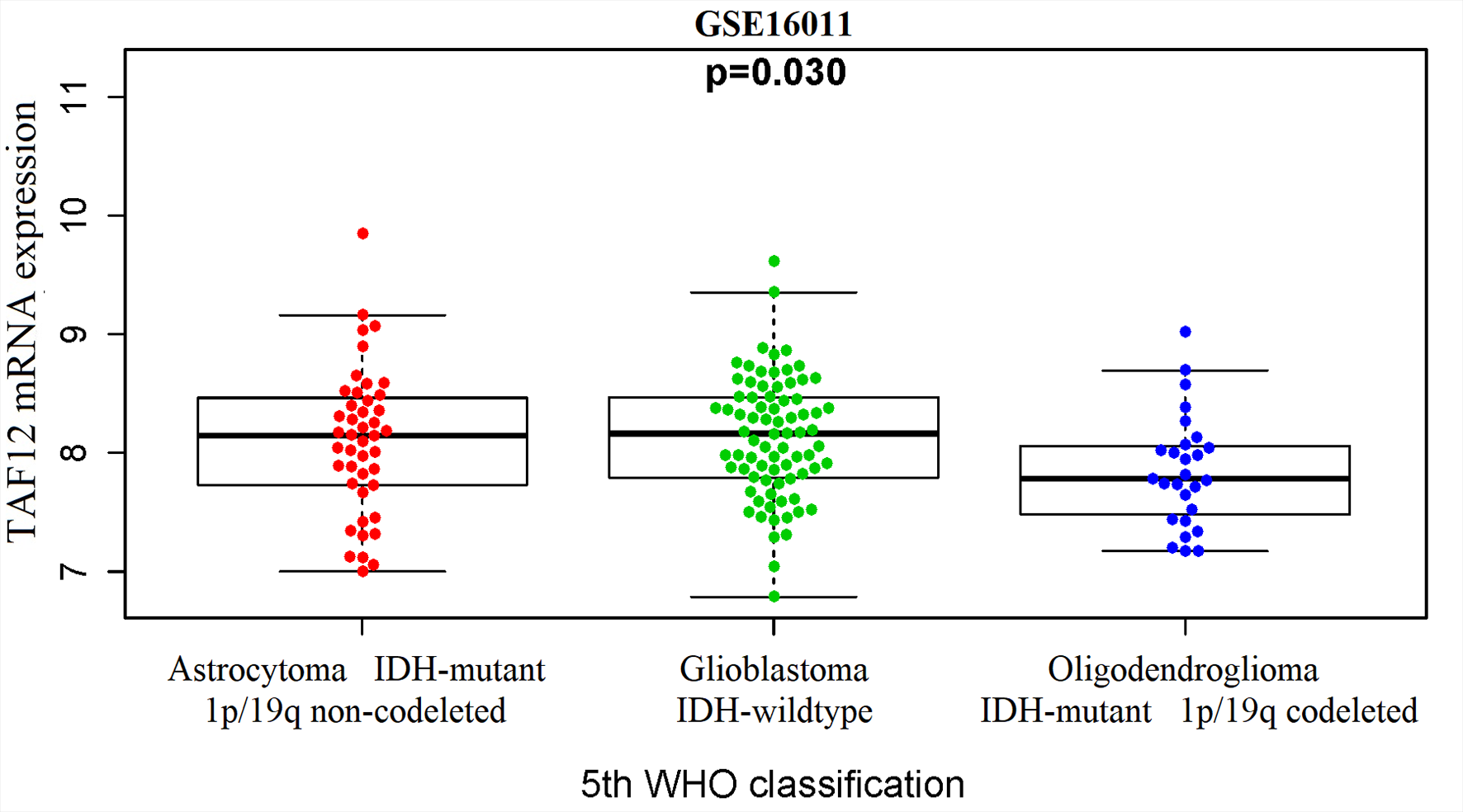

Supplement: Supplementary file 1 [file biomolecules-12-01847-s001.zip › supplementaryfigure S1C3.tif]

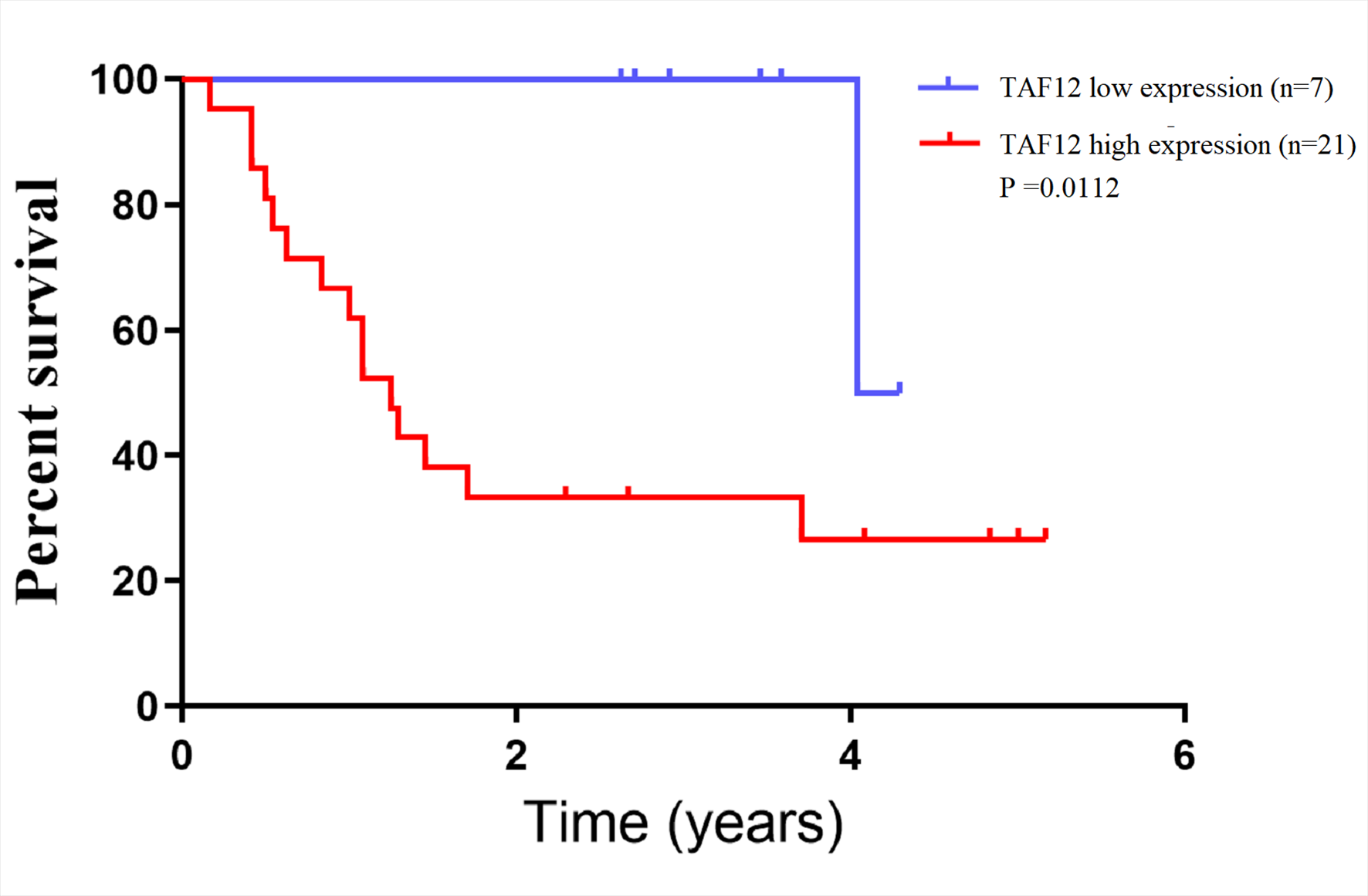

Supplement: Supplementary file 1 [file biomolecules-12-01847-s001.zip › supplementaryfigure S2.tif]
